# Joint effusion after anterior cruciate ligament reconstruction: Associations with higher postoperative physical activity, patella alta and increased quadriceps tension

**DOI:** 10.1002/jeo2.70678

**Published:** 2026-03-07

**Authors:** Jiebin Huang, Bin Song, Guohui Lin, Zilong He, Congda Zhang, Siu Ngor Fu

**Affiliations:** ^1^ Department of Rehabilitation Sciences The Hong Kong Polytechnic University Hong Kong SAR China; ^2^ Department of Sports Medicine Sun Yat‐sen Memorial Hospital of Sun Yat‐sen University Guang Zhou China; ^3^ Department of Sports Medicine The Sixth Affiliated Hospital of San Yat‐sen University Guang Zhou China; ^4^ Department of Radiology, Nanfang Hospital Southern Medical University Guang Zhou China

**Keywords:** ACL reconstruction, joint effusion, modifiable factor, muscle properties, quadriceps

## Abstract

**Purpose:**

(1) To explore postoperative factors associated with effusion after anterior cruciate ligament reconstruction (ACLR), and (2) to investigate the associations between effusion and quadriceps tension and activation across rehabilitation and return‐to‐sport (RTS) phases.

**Methods:**

In this cross‐sectional study, participants were assessed at approximately 3, 6, 12 and ~24 months post‐ACLR and further analysed by phase (3–6 vs. 12–24 months). Effusion (Anterior Cruciate Ligament Osteoarthritis Score [ACLOAS]) and patellar alignment (Insall‐Salvati ratio [ISR], bisect offset [BO], patellar tilt angle [PTA]) were quantified by 3.0‐T magnetic resonance imaging. Quadriceps tension and activation were quantified by shear wave elastography (SWE) and surface electromyography (EMG) during resting and isometric knee extension conditions. Physical activity was assessed using The International Physical Activity Questionnaire‐Short Form (IPAQ‐SF). Ordinal logistic regression tested associations with effusion; generalised linear models evaluated relationships between effusion and muscle outcomes (*α* = 0.05).

**Results:**

A total of 119 participants (75% male, age 29 ± 6 years) who underwent single‐bundle ACLR with hamstring tendon autograft were included in this study. Higher postoperative physical activity (MET‐min/week) was associated with higher effusion grade at 3‐month (odds ratio [OR] = 1.07, *p* < 0.01) and 24‐month (OR = 1.05, *p* = 0.05) groups; greater ISR was associated with higher effusion grade at ≥12 months (OR = 1.08, *p* < 0.01). Higher effusion grade was associated with higher vastus medialis and lateralis shear modulus at ≥12 months (*B* = 7–11 kPa, all *p* < 0.04) during the isometric knee extension condition; No association was observed for normalised EMG activation (all *p* > 0.09).

**Conclusions:**

Postoperative activity and patella alta were associated with joint effusion after ACLR. Additionally, residual effusion was associated with higher quadriceps tension under submaximal contraction beyond 1 year. These findings suggest that phase‐specific monitoring of physical activity and patellofemoral joint loading may benefit joint health and knee function after ACLR.

**Level of Evidence:**

Level IV.

## Graphical abstract

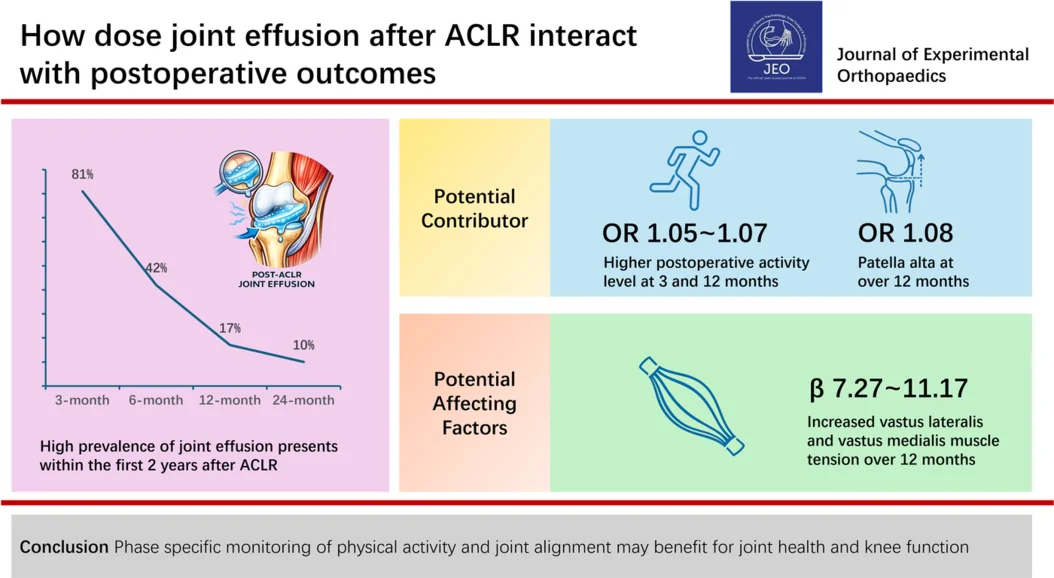

**Abbreviations**
ACL: anterior cruciate ligament
ACLR: anterior cruciate ligament reconstruction
AMI: arthrogenic muscle inhibition
ASIS: anterior superior iliac spine
BMI: body mass index
BO: bisect offset
DICOM: Digital Imaging and Communications in Medicine
EMG: electromyography
GLM: generalised linear model
IPAQ‐SF: International Physical Activity Questionnaire–Short Form
IQR: interquartile range
ISR: Insall–Salvati ratio
kgf: kilogram‐force
kPa: kilopascal (unit for shear modulus)
MET100: MET‐min·wk⁻¹ divided by 100 (scaling for modelling)
MET‐min·wk⁻¹: metabolic‐equivalent minutes per week
MRI: magnetic resonance imaging
MVC: maximal voluntary contraction
OA: osteoarthritis
OR: odds ratio
PFJ: patellofemoral joint
RF: rectus femoris
ROI; ROIs: region(s) of interest
RTS: return‐to‐sport
SD: standard deviation
SWE: shear‐wave elastography
VL: vastus lateralis
VM: vastus medialis
%MVC: percentage of MVC

## INTRODUCTION

After anterior cruciate ligament reconstruction (ACLR), joint effusion, excess synovial fluid within the knee joint capsule, remains common across rehabilitation [15, 24] and return‐to‐sport (RTS) phases [24, 35]. Effusion is clinically relevant because it is associated with quadriceps strength recovery, graft remodelling [14], re‐injury risk, and joint degeneration [15, 39] within the first 2 years after ACLR.

Higher pre‐injury activity levels have been associated with early postoperative effusion in some cohorts, suggesting a loading‐related component in effusion accumulation [14, 18]. However, whether postoperative physical activity is associated with effusion remains unclear. Excessive joint loading may contribute to synovial irritation and subsequent effusion. Patella alta, for example, has been associated with Hoffa's fat pad oedema [4, 40]. Patellar malalignment, such as patella alta and lateral displacement, is prevalent after ACL injury/reconstruction [17] and reflects altered patellofemoral joint (PFJ) loading patterns. These observations support a plausible association between patellar malalignment and joint effusion after ACLR.

Effusion is a well‐known contributor to arthrogenic muscle inhibition (AMI) [12], linking joint effusion to quadriceps weakness and incomplete activation. Furthermore, prior shear wave elastography (SWE) studies have demonstrated alterations in quadriceps muscle properties after ACLR [21, 23]. Yet, the relationship between joint effusion and quadriceps tension or activation has not been defined, and synovial reactivity appears to vary across recovery phases [15, 18, 24]. It remains unclear whether these associations are time‐specific.

Therefore, the primary aim of this study was to quantify the association of postoperative weekly physical activity (self‐reported physical activity over the previous 7 days) and MRI‐based measures of patellar malalignment with joint effusion at the time of assessment in individuals after ACLR. The secondary aims were: (1) to quantify the associations of joint effusion with quadriceps muscle tension and activation at rest and during submaximal isometric contraction; and (2) to determine whether these associations differed across postoperative time groups. It was hypothesised that (1) higher weekly physical activity and greater patellar malalignment would be associated with greater joint effusion; and (2) higher effusion grade would be associated with lower quadriceps tension and activation in a time‐specific manner.

## METHODS

### Study design

This cross‐sectional exploratory study was conducted at Sun Yat‐sen Memorial Hospital of Sun Yat‐sen University between February and October 2023. The protocol was approved by the Human Research Ethics Committee of The Hong Kong Polytechnic University (Ref. HSEARS20220523004) and prospectively registered with the Chinese Clinical Trial Registry (Ref. ChiCTR2200064220). Potentially eligible participants were identified from outpatient follow‐up clinics and were provided with a verbal explanation of the study together with a written information sheet describing the study aims, procedures, potential risks and benefits, and data confidentiality prior to any study procedures.

### Participants

Four groups of participants after primary ACLR were recruited according to time since surgery: (1) 3‐month group, assessed between 3 and 4 months post‐ACLR; (2) 6‐month group, 6–9 months post‐ACLR; (3) 12‐month group, 12–15 months post‐ACLR; and (4) 24‐month group, 19–26 months post‐ACLR. These timeframes were selected to align with commonly used rehabilitation and biological milestones after ACLR and to mirror previous outcome studies [14, 35, 43].

Inclusion criteria were: (1) age 18–40 years at the time of surgery; and (2) unilateral ACL injury with primary ACLR performed within 18 months after injury. Concomitant meniscal or cartilage injuries were permitted, including those managed at the index procedure (e.g. meniscal repair, partial meniscectomy and chondral debridement/chondroplasty).

Exclusion criteria were: (1) any additional ligament reconstruction or repair (e.g. posterior cruciate ligament, medial/lateral collateral ligament, posterolateral corner); (2) prior surgery on the ipsilateral knee; (3) prior contralateral ACLR; (4) subsequent knee injuries or re‐operations before the study visit; (5) absence or poor quality of postoperative MRI precluding reliable measurements; and (6) MRI contraindications (e.g., non‐MRI‐compatible implanted electronic devices, ferromagnetic aneurysm clips or foreign bodies, and severe claustrophobia).

### Surgical and rehabilitation background

All primary ACLR procedures were performed arthroscopically at three high‐volume orthopaedic centres in Guangzhou, China by fellowship‐trained surgeons, with most procedures (76%) performed at a single high‐volume centre. This study included patients who underwent primary ACLR between April 2021 and May 2023, met the eligibility criteria, and agreed to participate. A standardised single‐bundle ACLR using a four‐strand hamstring autograft (semitendinosus/gracilis) was performed in all cases. The femoral tunnel was created arthroscopically using an anteromedial portal technique, with the guide pin positioned at the anatomical ACL footprint on the medial wall of the lateral femoral condyle and the tunnel reamed to the graft diameter (8–9 mm) with EndoButton suspensory fixation (Smith & Nephew, Watford, UK). Concomitant meniscal tears were treated with partial meniscectomy and/or meniscal repair, as indicated. The Hoffa's fat pad was generally preserved, with only limited resection when it obstructed arthroscopic visualisation or instrument passage. Passive full knee range of motion was confirmed intraoperatively before graft fixation.

All participants were instructed to follow a similar, phase‐based postoperative rehabilitation programme. For isolated ACLR, early weight bearing as tolerated was encouraged from the first postoperative week, whereas patients with meniscal repair were non‐weight‐bearing for 4 weeks and progressed to full weight bearing by week 6. Rehabilitation focused on early restoration of full extension and 0°–90° flexion within the first 4 weeks, followed by progressive neuromuscular retraining. Straight‐line jogging and low‐impact sport‐specific drills were introduced no earlier than 3 months postoperatively, and unrestricted competitive sports were generally permitted at approximately 10–12 months once basic return‐to‐sport criteria (adequate strength, dynamic stability and no giving‐way episodes) were met.

### Demographic and surgical information

Demographic data (age, sex and body mass index [BMI]) and surgical details (time from injury to surgery [>4 weeks defined as delayed surgery] [19], graft type and meniscal management) were extracted from medical records. Meniscal management was categorised as: none, unilateral repair (with or without partial meniscectomy), or bilateral repair (with or without partial meniscectomy).

### Study procedure

Participants attended two visits within 2 weeks (mean interval 3.7 ± 3.3 days) at a single postoperative time point corresponding to their allocated group. All measurements were performed in a seated position with 30° knee flexion, 15° hip flexion and neutral rotation, reflecting the angle of peak loading during stair descent [5] and a commonly used rehabilitation milestone [6, 30].

On Day 1, maximum voluntary contraction (MVC), quadriceps tension and quadriceps activation were assessed. MVC was measured using a handheld dynamometer (MicroFET 2, Hoggan Scientific, LLC, USA) mounted on a custom frame; three MVC trials were performed and the peak value (kilogram‐force, kgf) recorded. Quadriceps tension and activation were then measured at rest and during isometric knee extension at 15% MVC, with submaximal load chosen following McPherson et al. to maintain shear modulus within the measurable range [21]. A custom non‐metallic loading apparatus was used to maintain isometric contractions (Figure in Supporting Information: File S1). On Day 2, knee MRI was obtained in the radiology unit.

### Outcome measurements

#### Joint effusion

**Figure 1:**
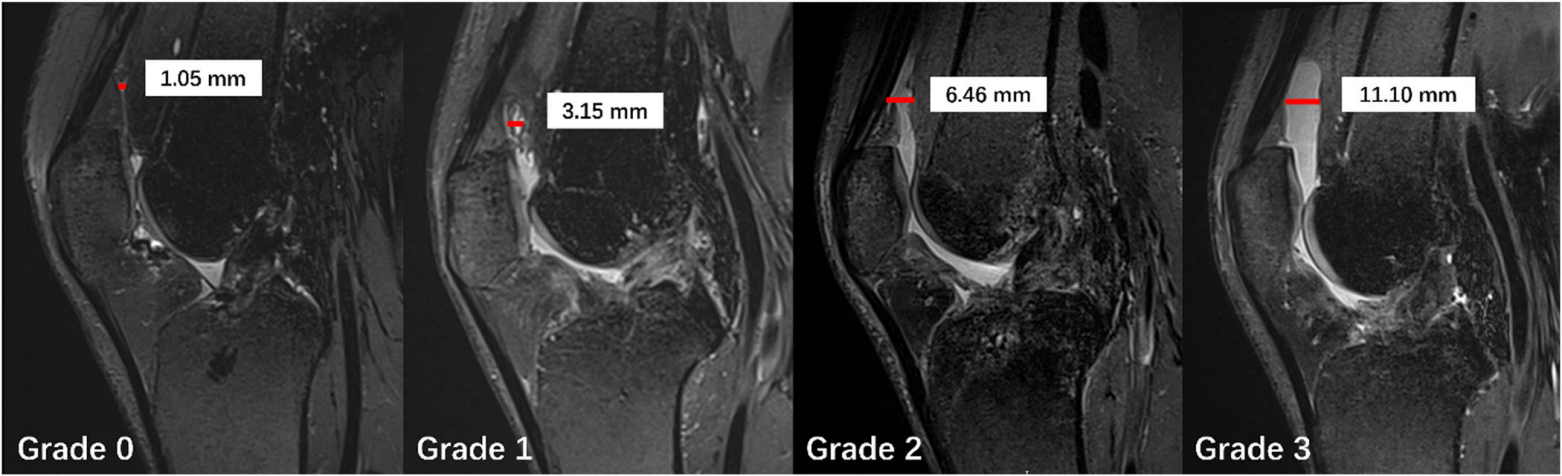
Joint effusion classification from Grade 0 to Grade 3, according to semi‐quantitative tool Anterior Cruciate Ligament Osteoarthritis Score (ACLOAS). The scoring is according to the capsular distension thickness: 0 (<2 mm), 1 (≥2 and <5 mm), 2 (≥5 and <10 mm) and 3 (≥10 mm).

MRI assessments were conducted using a 3.0 T unit equipped with an 18‐channel knee coil (MAGNETOM Skyra, Siemens Healthcare, Erlangen, Germany). The MRI protocol used a three‐dimensional T2‐weighted SPACE FS sequence (repetition time = 1200 ms; echo time = 117 ms; slice thickness = 0.35 mm). Joint effusion was evaluated using the Anterior Cruciate Ligament Osteoarthritis Score (ACLOAS) [28], which classifies capsular distension thickness as Grade 0 (<2 mm), 1 (≥2 and <5 mm), 2 (≥5 and <10 mm) or 3 (≥10 mm) (Figure 1). Effusion grading was independently performed by an experienced musculoskeletal radiologist. Intra‐rater reliability was assessed on a randomly selected subset of 20 MRIs that were re‐graded after a 1‐week washout period, with the rater blinded to the initial ratings and clinical information. Reliability was excellent (ICC (3,1) = 0.95), calculated using a two‐way mixed‐effects model with absolute agreement and single‐measure definition.

#### Patellar alignment

**Figure 2:**
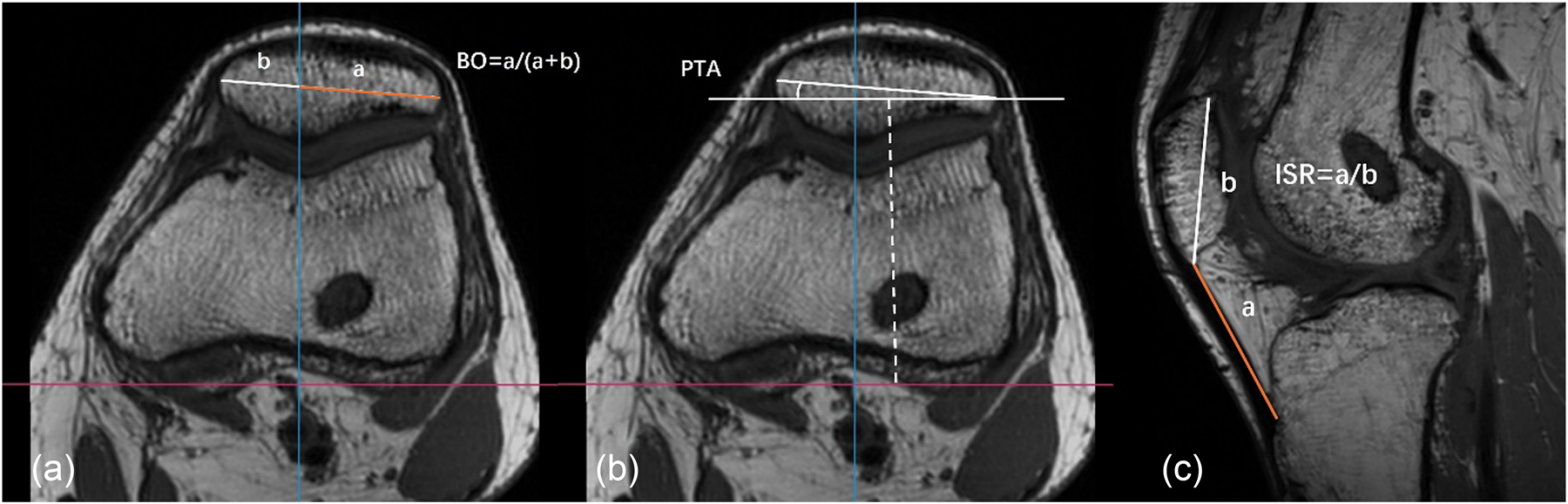
Patellar alignment including: (a) Bisect offset (BO), represents the proportion of lateral displacement relative to midline as a percentage of total patellar width, calculated as a/(a + b), where a is the distance from the trochlear midline to the lateral edge of the patella and b is the distance from the trochlear midline to the medial edge of the patella. (b) Patellar tilt angle (PTA), denotes the angle formed between the posterior condylar line and a line defining maximal patellar width; and (c) Insall–Salvati ratio (ISR), signifies the ratio between patellar tendon length and maximum length of patella.

Patellar alignment was assessed on three‐dimensional T1‐weighted SPACE sequence (repetition time = 400 ms; echo time = 10 ms; slice thickness = 0.36 mm). Measurements were performed using RadiAnt DICOM Viewer (Medixant, Poland) by a trained research student under the supervision of an experienced musculoskeletal radiologist. Intra‐rater reliability ranged from 0.79 to 0.87 (ICC (3,1), *n* = 20, 1‐week washout), and inter‐rater reliability ranged from 0.72 to 0.88. Inter‐rater ICCs were calculated using a two‐way mixed‐effects model with absolute agreement and single‐measure definition (ICC (3,1), *n* = 20). This approach was selected to quantify agreement between the two specific raters involved in the present study rather than to generalise to other raters. Bisect offset (BO), patellar tilt angle (PTA) and the Insall–Salvati ratio (ISR) were measured on axial and sagittal images (Figure 2) [20]. ISR was multiplied by 100% for modelling.

#### Quadriceps tension and activation

**Figure 3:**
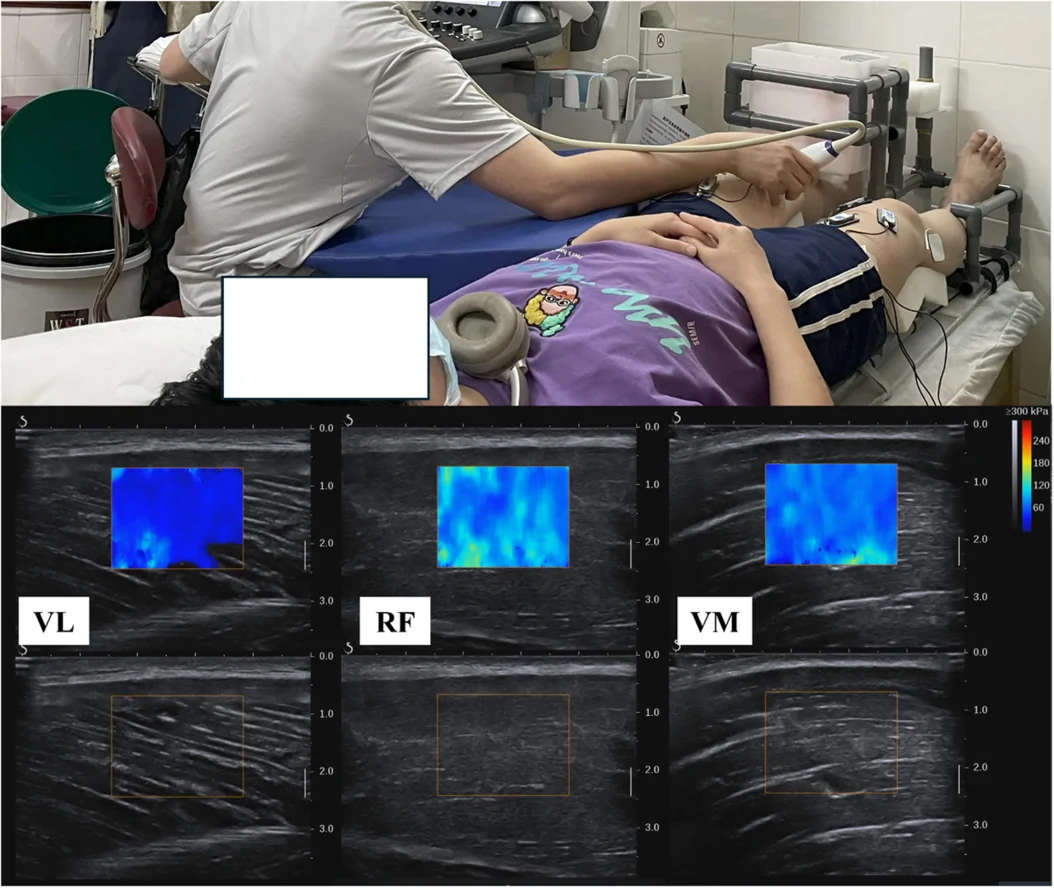
A typical image of quadriceps muscle shear modulus under 15% of maximum voluntary contraction. RF, rectus femoris; VL, vastus lateralis; VM, vastus medialis.

Quadriceps tension was measured using shear wave elastography (Aixplorer Version 4.2, Supersonic Imagine, France) of the vastus lateralis (VL), vastus medialis (VM) and rectus femoris (RF), following previously described protocols [42]. Shear modulus (kPa) was quantified from colour‐coded elastography maps by averaging a 5‐s window in each recording (Figure 3), with regions of interest drawn to exclude artefacts.

Quadriceps activation was measured by surface electromyography (EMG, B&L Engineering, Inc, USA). Electrodes were placed adjacent to SWE probe sites after standard skin preparation (Figure in the supplemental file). EMG signals were processed and normalised to the peak value during MVC, and expressed as %MVC, by using Noraxon MR 3.16 (Noraxon USA).

#### Physical activity level

Postoperative weekly physical activity was assessed using the International Physical Activity Questionnaire‐Short Form (IPAQ‐SF), which records walking, moderate and vigorous activities during the previous 7 days [3]. Total activity was converted into MET‐minutes per week according to IPAQ scoring guidelines and divided by 100 (MET100) for regression analyses [3].

### Statistical analyses

Analyses were conducted in SPSS v24 and R v4.3.1 (two‐tailed *α* = 0.05). Normality was assessed with the Shapiro–Wilk test. Continuous data are reported as mean ± SD or median [IQR], and categorical data as *n* (%). Continuous and ordinal variables were compared across the four postoperative time‐since‐surgery groups using analysis of variance or Kruskal–Wallis tests; categorical variables were compared using *χ*² or Fisher's exact tests.

To identify factors associated with effusion, ordinal logistic regression (enter method) was performed separately in each group, with ACLOAS effusion grade as the dependent variable. Predictors were physical activity level (MET100) and patellar alignment indices (BO, PTA, ISR). Covariates were selected a priori from the literature and included age at surgery, delayed surgery and meniscal management [15, 18, 24].

To evaluate the impact of effusion on quadriceps properties, generalised linear models (GLM) were used, with quadriceps tension and activation as dependent variables and ACLOAS effusion grade as the independent variable, adjusted for age at surgery and sex. A Gaussian identity link was used when residuals were approximately normal; otherwise, a Gamma distribution with log link was applied.

Because unrestricted, sport‐specific activity is not recommended until at least 9–12 months after ACLR [16], the four postoperative groups were collapsed into two phases: the rehabilitation phase (3–6 months) and the RTS phase (12–24 months). Effusion grade differences between phases were examined with Mann–Whitney *U* tests.

A priori sample size was calculated using G*Power 3.1 for linear regression, assuming 80% power, *α* = 0.05, effect size 0.22 and three predictors (based on pilot data from prior study), yielding a target of 54 participants per group (total *n* = 216). Post hoc power was also evaluated for the main analyses.

## RESULTS

**Figure 4:**
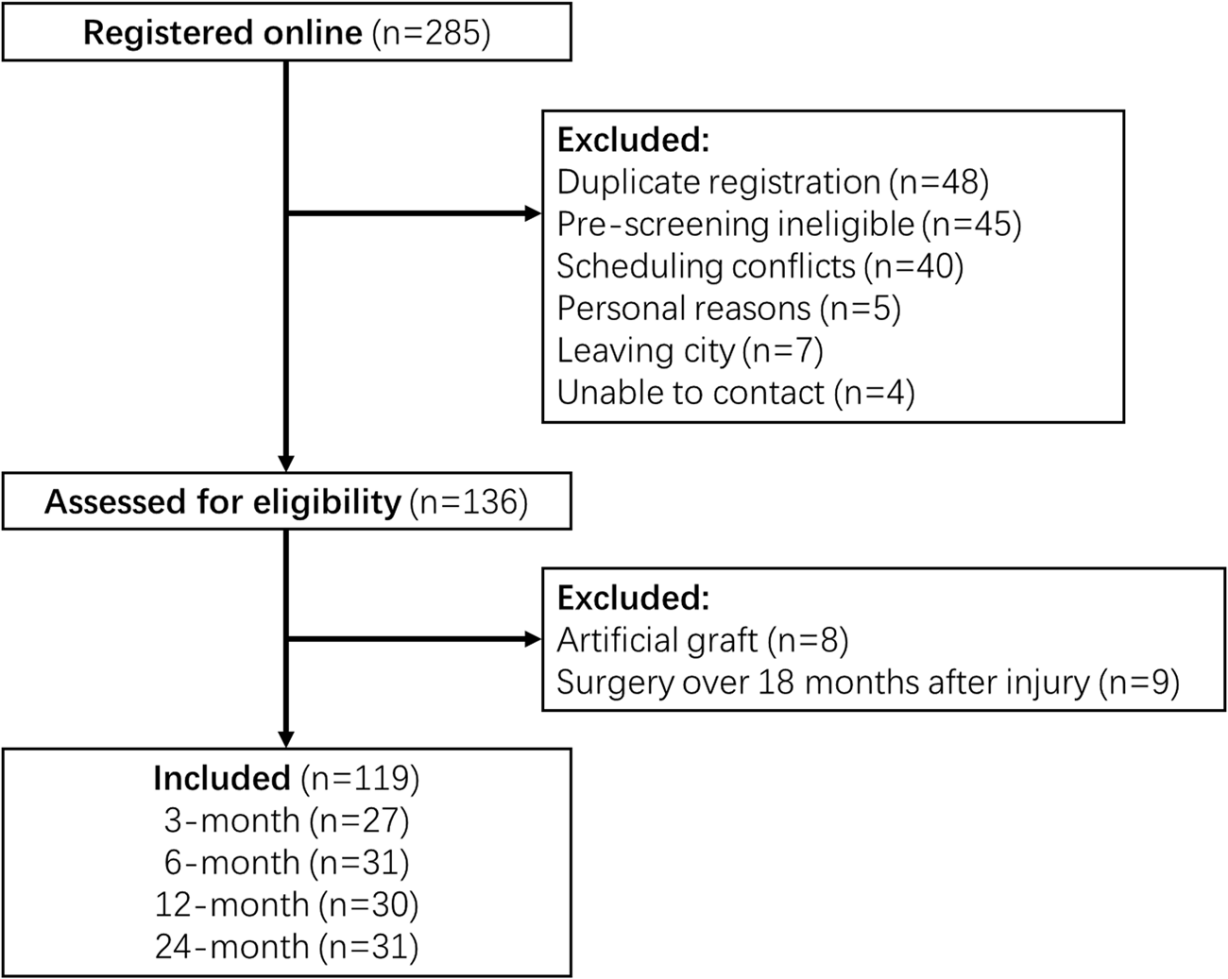
Study flow diagram.

A total of 285 individuals registered their interest in participating in the study. Of these, 149 did not proceed to hospital assessment due to duplicate registration (*n* = 48), failing pre‐screening eligibility based on the online registration form (*n* = 45), scheduling conflicts (*n* = 40), personal reasons (*n* = 5), relocation out of the study city (*n* = 7), or inability to contact (*n* = 4) (Figure 4). The remaining 136 individuals attended the hospital visit and completed MRI and clinical assessments. After full eligibility assessment, 17 participants were excluded (artificial graft: *n* = 8; surgery performed >18 months after injury: *n* = 9), leaving 119 participants for analysis across the four postoperative groups (75% male, age 29 ± 6 years, BMI 24.2 ± 3.1 kg/m², 10% with cartilage lesion).

Among participants with meniscal injury, medial meniscal tears were present in 89% (body 17%, posterior horn 58%, other locations 15%), and lateral meniscal tears in 78% (body 21%, posterior horn 35%, other locations 22%). Meniscal management at the index procedure included meniscal repair in 94% of knees with a meniscal tear, and in 47% of these cases repair was combined with partial meniscectomy. Details of meniscal management are summarised in Table 1. Age, BMI, sex and meniscal management did not differ among groups (Table 1). Descriptive data of patellar alignment, quadriceps strength, tension and activation are presented in Table 2.

**Table 1:** Patient demographics of each group.

	3‐month (*n* = 27)	6‐month (*n* = 31)	12‐month (*n* = 30)	24‐month (*n* = 31)	*p*‐value
Time post‐surgery, months	3.2 ± 0.6	7.1 ± 1.1	12.6 ± 1.0	21.2 ± 2.1	
Age, years	28 ± 6	30 ± 6	28 ± 6	28 ± 5	0.78
Male, *n* (%)	20 (74%)	18 (58%)	25 (83%)	26 (84%)	0.07
BMI, kg/m²	23.7 ± 2.5	23.8 ± 3.0	24.5 ± 3.9	24.6 ± 3.0	0.68
MET/100, median (IQR)	23.5 (10.1–54.4)	33.1 (11.8–63.5)	18.7 (9.4–29.5)	24.8 (12.2–37.3)	0.28
Delayed surgery, *n* (%)	10 (37%)	13 (42%)	13 (43%)	13 (42%)	0.98
Meniscal management, *n* (%)					0.60
None	0 (0%)	1 (3%)	2 (7%)	3 (10%)	
Unilateral	5 (19%)	8 (26%)	5 (17%)	6 (19%)	
Lateral	1 (20%)	0 (0%)	1 (20%)	3 (50%)	
Medial	4 (80%)	8 (100%)	4 (80%)	3 (50%)	
Bilateral	22 (82%)	22 (71%)	23 (77%)	22 (71%)	
Cartilage lesion, *n* (%)					0.64
Any lesion	3 (11%)	3 (11%)	4 (13%)	2 (7%)	
Patellar	0 (0%)	1 (3%)	0 (0%)	2 (7%)	
Femoral	3 (11%)	3 (10%)	4 (13%)	2 (7%)	
Tibial	0 (0%)	3 (10%)	0 (0%)	0 (0%)	
Joint effusion, *n* (%)					**<0.01**
0	3 (11%)	7 (23%)	11 (37%)	19 (61%)	
1	2 (7%)	11 (36%)	14 (47%)	9 (29%)	
2	16 (59%)	10 (32%)	5 (17%)	3 (10%)	
3	6 (22%)	3 (10%)	0 (0%)	0 (0%)	

*Note*: Data with *p* < 0.05 are **bolded**.

Abbreviations: BMI, body mass index; IQR, interquartile range; kg, kilogram; MET/100, MET‐min∙wk⁻¹/100.

**Table 2:** Descriptive data of patellar alignment, quadriceps strength, tension and activation.

	3‐month (*n* = 27)	6‐month (*n* = 31)	12‐month (*n* = 30)	24‐month (*n* = 31)	*p*‐value
Patellar alignment					
ISR (%)	106.93 ± 16.42	107.19 ± 14.54	108.06 ± 13.42	106.67 ± 13.52	0.99
BO (%)	59.76 ± 5.30	59.33 ± 4.64	59.90 ± 5.52	59.36 ± 4.18	0.96
PTA (°)	6.16 ± 3.61	6.84 ± 4.08	5.97 ± 3.84	6.49 ± 3.46	0.81
Quadriceps strength					
MVC (kgf)	30.88 ± 9.28	32.94 ± 11.65	35.03 ± 11.61	41.96 ± 10.64	<0.01
BW% (%)	0.59 ± 0.11	0.60 ± 0.14	0.53 ± 0.17	0.59 ± 0.14	0.25
Quadriceps tension					
VL_T_ (kPa)	2.99 ± 0.53	3.15 ± 0.58	3.13 ± 0.53	3.33 ± 0.55	0.15
RF_T_ (kPa)	3.11 ± 0.53	3.25 ± 0.63	3.22 ± 0.68	3.15 ± 0.45	0.80
VM_T_ (kPa)	3.07 ± 0.57	3.19 ± 0.57	3.32 ± 0.69	3.53 ± 0.61	0.04
Quadriceps activation					
VL_E_ (%)	17.72 ± 8.11	15.29 ± 8.19	19.19 ± 9.33	13.77 ± 6.88	0.05
RF_E_ (%)	11.84 ± 6.02	9.39 ± 6.62	9.21 ± 5.61	9.43 ± 3.75	0.26
VM_E_ (%)	9.87 ± 4.18	11.10 ± 7.68	12.13 ± 7.88	9.31 ± 4.83	0.33

Abbreviations: BO, bisect offset*100%; BW%, MVC normalised by body weight; _E_, value of normalised activation; ISR, Insall–Salvati ratio*100%; kgf, kilogram‐force; MVC, maximal voluntary contraction value; PTA, patellar tilt angle; RF, rectus femoris; _T_, value of shear modulus (tension); VL, vastus lateralis; VM, vastus medialis.

Effusion significantly decreased across time groups (*p* < 0.01). Marked effusion (Grade 2 and 3) was observed in 81% in the 3‐month group but 10% in the 24‐month group, whereas the proportion without effusion rose from 11% to 61% (Table 1). Pairwise differences were significant between 3‐ and 6‐month groups (*p* < 0.01), 12‐ and 24‐month groups (*p* = 0.04), and between the Rehab (3–6 months) and RTS (12–24 months) phases (*p* < 0.01).

### Factors associated with joint effusion

Table 3 illustrates that higher physical activity was related to higher effusion grade in 3‐month (OR = 1.07, *p* < 0.01, power = 93%) and 24‐month groups (OR = 1.05, *p* = 0.05, power = 55%). Patella alta (ISR) was also associated with higher effusion in 24‐month group (OR = 1.18, *p* < 0.01, power = 93%) and across the RTS phase (OR = 1.08, *p* < 0.01, power = 97%). BO and PTA were not significantly associated with effusion grades.

**Table 3:** Associations between contributors and joint effusion.

	Time & Phase group, OR (95% CI)					
Factor	3‐month	6‐month	Rehab phase (3–6 months)	12‐month	24‐month	RTS phase (12–24 months)
Male	0.59 (0.10, 3.66)	0.40 (0.10, 1.69)	0.62 (0.22, 1.76)	**0.13 (0.02, 1.00)**	3.10 (0.22, 43.53)	0.48 (0.13, 1.81)
BMI	1.19 (0.80, 1.78)	0.96 (0.78, 1.18)	0.98 (0.82, 1.18)	0.86 (0.71, 1.05)	0.97 (0.73, 1.30)	0.90 (0.77, 1.05)
MET/100	**1.07 (1.02, 1.12)**	0.99 (0.97, 1.01)	1.00 (0.98, 1.02)	1.01 (0.99, 1.04)	**1.05 (1.00, 1.10)**	1.02 (1.00, 1.04)
ISR	0.99 (0.95, 1.05)	1.00 (0.95, 1.06)	1.00 (0.97, 1.04)	1.03 (0.98, 1.09)	**1.18 (1.06, 1.32)**	**1.08 (1.03, 1.13)**
BO	0.99 (0.84, 1.15)	0.96 (0.82, 1.13)	1.03 (0.93, 1.14)	0.97 (0.85, 1.12)	0.92 (0.75, 1.14)	0.99 (0.89, 1.11)
PTA	1.08 (0.85, 1.36)	0.95 (0.78, 1.15)	1.01 (0.88, 1.16)	1.10 (0.91, 1.33)	0.87 (0.67, 1.12)	1.00 (0.87, 1.15)

*Note*: Analyses adjusted for age, meniscal management and early/delayed surgery. Data with *p* < .05 are **bolded**.

Abbreviations: BO, bisect offset*100%; ISR, Insall–Salvati ratio*100%; MET/100, MET‐min∙wk⁻¹/100; PTA, patellar tilt angle; Rehab, rehabilitation; RTS, return‐to‐sport.

### Associations between joint effusion and muscle tension/activation

**Figure 5:**
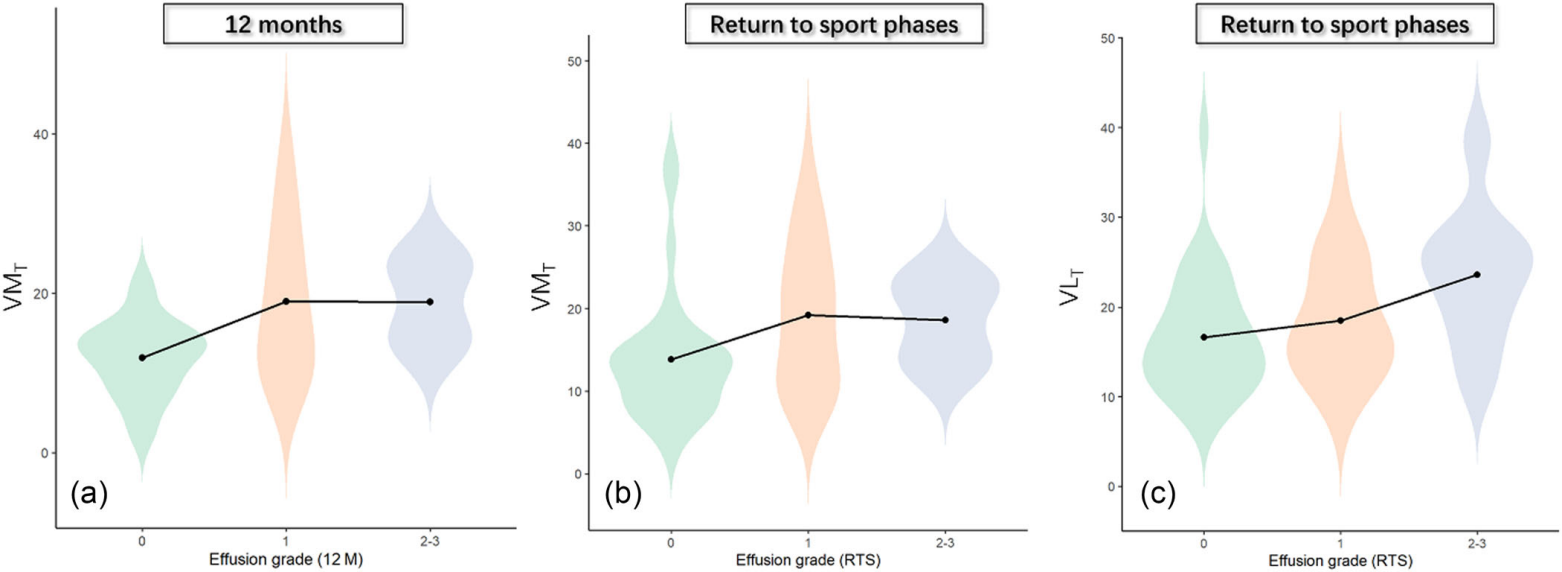
Distribution of quadriceps tension across effusion grades. (a) Vastus medialis tension distribution during isometric knee extension condition in 12‐month group across effusion grades. (b) Vastus medialis tension distribution during isometric knee extension condition in return‐to‐sport phase across effusion grades. (c) Vastus lateralis tension distribution during isometric knee extension condition in return‐to‐sport phase across effusion grades. The **black dots** represent the mean tension at each effusion grade, and the black line connects these means to highlight the trend across increasing effusion severity.

Results of univariate analysis between effusion and quadriceps strength, tension and activation are provided in the supplemental file. After adjusting age and sex, multivariable GLM analyses found that higher effusion grade was linked to higher VM tension in 12‐month group (*B*  =  9.19 and 11.17 for Grades 1 and 2 vs. 0, *p* = 0.01) and in the RTS phase (*B*  =  0.34 and 0.36 for Grades 1 and 2 vs. 0, *p* = 0.04). Higher effusion grade was also related to higher VL tension in RTS phase (*B*  =  7.27, *p* = 0.03) (Figure 5 and Table 4). EMG normalised activation showed no association with effusion (all *p* > 0.09).

**Table 4:** Generalised linear models analyses between joint effusion and quadriceps tension/activation.

Outcomes	Effusion 1 *B* (95% CI)	Effusion 2 *B* (95% CI)	Overall *χ*²	*p*‐Overall	Power
VM_T_ (12‐month)^b^	**9.19 (3.93–14.45)**	**11.17 (4.21–18.13)**	12.78	**0.01**	86%
VM_T_ (RTS phase)^a^	**0.34 (0.09–0.59)**	**0.36 (0.02–0.71)**	10.15	**0.04**	77%
VL_T_ (RTS phase)^b^	1.69 (−1.91, 5.28)	**7.27 (2.35–12.18)**	11.03	**0.03**	81%

*Note*: Generalised linear models were fitted using either a Gaussian identity link or a Gamma distribution with log link, as indicated. Reference category = Effusion 0. Overall *p* values come from Type III Wald *χ*² tests. Analyses adjusted by age and sex. Data with *p* < .05 are **bolded**.

Abbreviations: CI, confidence interval; GLM, generalized linear model; RTS, return‐to‐sport; VM_T_, tension of vastus medialis during contraction.

^a^ Using Gamma‐Log GLM.

^b^ Using Linear GLM.

## DISCUSSION

The most important finding of this study was identified two phase‐specific factors associated with joint effusion after ACLR, whereby higher effusion grade was associated with higher reported activity at 3 and 24 months, and with patella alta at ≥12 months. In addition, at ≥12 months, higher effusion grade was associated with greater quadriceps shear modulus during standardised isometrics, whereas surface EMG amplitude showed no parallel association.

### Post‐ACLR effusion

The prevalence of moderate‐to‐severe effusion was reduced accordingly in 3‐, 6‐, 12‐ and 24‐month groups. The early prevalence in this study was higher than some prior reports [15] but comparable at later time‐point groups [24, 35]. One possible explanation is the higher proportion of concomitant meniscal procedures in this study (>90%) compared with previous studies (~60%) [15, 24], which has been linked to early postoperative effusion in prior work [18]. In addition, focal cartilage lesions were present in a minority of participants in this study, which may also contribute to synovial inflammation and persistent effusion. Clinically, a high early prevalence of effusion may align with greater synovial reactivity in the postoperative environment [9], which may increase the risk of chondral degeneration [26, 29].

### Postoperative activity and patellar alta were associated with joint effusion

An association between higher postoperative physical activity (at 3‐ and 24‐months post‐ACLR) and joint effusion was observed in this study. Although self‐reported activity has recognised limitations, prior cellular studies show that synovial environment is sensitive to mechanical stimuli [32]. Upregulating inflammatory mediators under high cyclic loading increases endothelial permeability and recruits inflammatory cells, which is a possible link to effusion. In addition, although patients usually returned to sport over 1 year post‐surgery, prior work showed that residual effusion was still found in 87% of patient at 2 years post‐ACLR and associated with asymmetric loading in gait [7]. Higher physical activity may amplify gait loading asymmetry, which may contribute to residual effusion.

Patella alta was also associated with higher effusion grade at ≥12 months. A likely reason is that patella alta after ACL injury/ACLR reflects altered PFJ stress, which could promote joint irritation and effusion, especially during higher‐impact activity [8, 41]. The reported association between patella alta and superolateral Hoffa's fat pad oedema in people with patellofemoral pain, in young adults, and in adolescent athletes without knee pain might partly support the notion of PFJ overload/inflammatory changes in the presence of patella alta [4, 36, 40]. Although Hoffa's fat pad is an extra‐synovial tissue, it is vascular and immunologic activity adjacent to synovium [44]. Mechanical irritation of this richly innervated, vascularises region may trigger pro‐inflammatory cytokines (e.g., IL‐6 and TNF‐α), promoting synovial‐fluid production and a chronic effusion phenotype [4]. After returning to sport, most patients resume running and squatting, which can induce repeated impingement in individuals with patella alta [4].

Taken together, excessive loading, either from participation in physical activity or from abnormal knee loading, were associated with joint effusion in both the early and RTS phase after ACLR. These findings suggest that surgeons may help to reduce postoperative effusion by counselling patients to avoid early high‐impact or high‐volume activities and by closely monitoring and addressing patellofemoral malalignment (e.g. patella alta) through timely referral for targeted rehabilitation.

### Impacts of joint effusion on muscle properties and activation

Although joint effusion induced AMI is well known in laboratory and clinical studies, whether effusion is associated with quadriceps mechanical tension and neural activation in patients over 6 months post‐ACLR remains unclear. This study shows that higher effusion grade was associated with greater shear modulus in the vastus medialis and lateralis during standardised submaximal (15% MVC) contractions over 1 year post‐ACLR, but no association was found in muscle activation. A greater shear modulus during a given low‐level contraction indicates that, for patients, these muscles are operating in a mechanically stiffer state (i.e., higher intramuscular tension) to produce the same relative torque [1]. For patients, it may reflect a less efficient neuromuscular strategy and potentially greater perceived muscle fatigue and patellofemoral joint loading during daily tasks. This finding is inconsistent with previous laboratory studies that acute effusion induces quadriceps inhibition [25, 38]. However, Jones et  al. could not detect an association between effusion size and muscle inhibition in patients with more than 6 months of swelling [13], and Rutherford et  al. later reported that knee OA patients with chronic effusion had greater quadriceps activation than those without [31]. Taken together, these results suggest that the influence of chronic effusion on the quadriceps may shift from acute inhibition towards a compensatory over‐recruitment pattern. Consistent with this, prior studies have reported greater motor unit recruitment [33, 37] and action potentials [34] of VM during submaximal contraction (30%–70% MVC) in patients 1–2 years post‐ACLR. One possible explanation relates to compensatory adaptation in the central nervous system [22, 27]. As chronic effusion may increase active motor threshold, it requires greater primary motor cortex activation to sustain quadriceps contraction [33], which in turn manifests as higher shear modulus during submaximal tasks.

Interestingly, higher effusion grade was associated with greater quadriceps tension but not with EMG amplitude beyond 12 months post‐ACLR. The reason for this may relate to the different recovery trajectories of these two factors. Muscle activation measured by EMG is nearly recovered at 1 year post‐ACLR [2], whereas muscle tension measured by SWE remains elevated at this stage [21]. Muscle tension reflects both neural activation and structural adaptation and is closely related to muscle force [10, 11]. Therefore, interventions aimed at accelerating effusion resolution should be explored in future longitudinal trials to determine whether they are benefit to muscle tension normalisation and strength recovery in the RTS phase after ACLR.

Additionally, no association was found between effusion and muscle tension/activation during the early postoperative phase. These findings contrast with Johnson et al. (2024) [12], who reported a slight negative association between effusion volume and quadriceps voluntary activation (VA) at 4 months post‐ACLR. Unlike that study, this study captured muscle activation at only 15% MVC. At such a low load, the requirement can be met by recruiting low‐threshold motor units, so the same central inhibition may be functionally compensated. This might explain the difference in findings.

### Limitations

This study has several limitations. First, there was substantial attrition between online registration and hospital assessment, mainly due to ineligibility and logistical constraints, which may have introduced selection bias and limit generalisability. Future studies should confirm these associations in larger cohorts. Second, quadriceps properties were assessed only during low‐intensity isometric contractions at a single site per muscle; given known intra‐muscular heterogeneity, these SWE/EMG measures may not fully reflect whole‐muscle behaviour or performance at higher force levels or during dynamic tasks. Third, postoperative physical activity was assessed using the IPAQ‐SF, a self‐reported questionnaire that captures overall weekly activity (including walking and rehabilitation exercises) but is less sensitive to sport‐specific loading, particularly in the early postoperative phase. Fourth, although a standardised anteromedial portal technique was used for femoral tunnel placement, tunnel position or rotatory knee instability were not quantitatively assessed; thus, a modest degree of residual confounding from surgical variation cannot be excluded. Fifth, preoperative MRI and range of motion at the time of surgery were not recorded in a standardised manner; therefore, these baseline clinical factors could not be adjusted for in the analyses. Future studies should prospectively collect standardised preoperative and perioperative measurements of effusion and range of motion to better account for baseline status. Finally, joint effusion was graded using the semi‐quantitative ACLOAS scale rather than volumetric measurements, which may have reduced sensitivity to small differences in fluid volume. Future studies might consider volumetric measures to enhance sensitivity.

## CONCLUSION

This study indicated that physical activity and patellar alignment relate to post‐ACLR effusion in rehabilitation and RTS phases. Joint effusion is associated with greater quadriceps tension during the RTS phase. Further studies should evaluate phase‐specific strategies for controlling post‐ACLR effusion and its impact on quadriceps properties.

## AUTHOR CONTRIBUTIONS

Jiebin Huang and Siu Ngor Fu responsible for the conception and design of the study. Bin Song and Siu Ngor Fu responsible for the resources and subject recruitment of this study. Jiebin Huang, Guohui Lin and Congda Zhang were responsible for data collection. Jiebin Huang and Zilong He were involved in the processing and statistical analysis of data. Jiebin Huang, Bin Song and Siu Ngor Fu were involved in the drafting of the manuscript; and all authors contributed to the interpretation of the data for the work and revising it critically for important intellectual content. All the authors finally approved the manuscript. Siu Ngor Fu was responsible for obtaining project funding and takes responsibility for the integrity of the work as a whole. All authors have read and agreed to the published version of the manuscript.

## CONFLICT OF INTEREST STATEMENT

The authors declare no conflicts of interest.

## ETHICS STATEMENT

Ethical approval was granted by the Human Research Ethics Committee at The Hong Kong Polytechnic University (reference number HSEARS20220523004). All participants provided written informed consent.

## DECLARATION

ChatGPT 5 thinking model was used only for language polishing and formatting, and all authors reviewed and edited the content and took full responsibility for it.

## Supporting information

Supplemental_file.

## Data Availability

Data available on request from the authors.
