# Supplementary material for: Joint effusion after anterior cruciate ligament reconstruction: Associations with higher postoperative physical activity, patella alta and increased quadriceps tension
Source: J Exp Orthop. 2026 Mar 7;13(1):e70678. doi: 10.1002/jeo2.70678 (PMC12966962; doi:10.1002/jeo2.70678)
Supplement: Supplementary file 1 — Supplemental_file. [file JEO2-13-e70678-s001.docx]

**Supplementary**


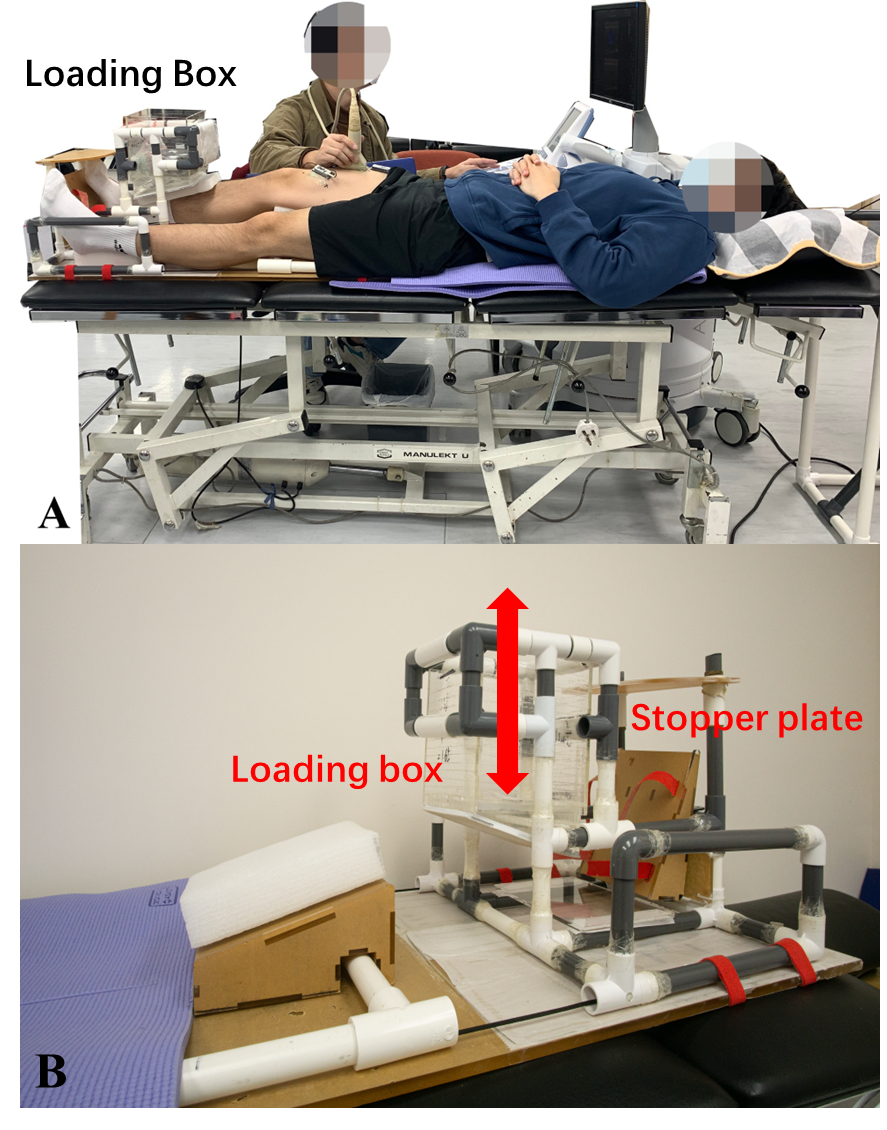


FIGURE S1. Loading platform for isometric contraction using in quadriceps assessments.


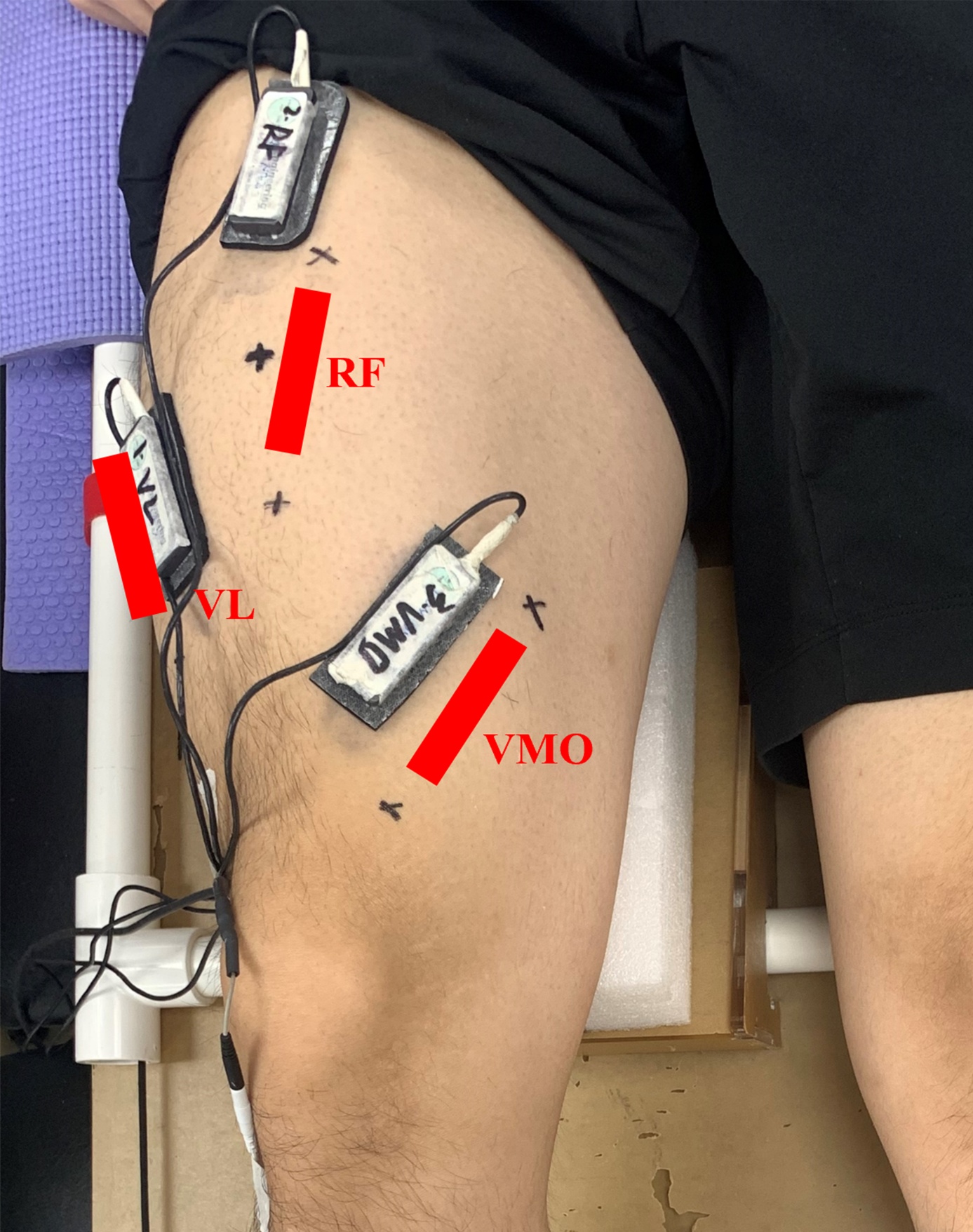


FIGURE S2. Locations of shear wave elastography probe (Red block) and electrodes for vastus lateralis, rectus femoris and vastus medialis.


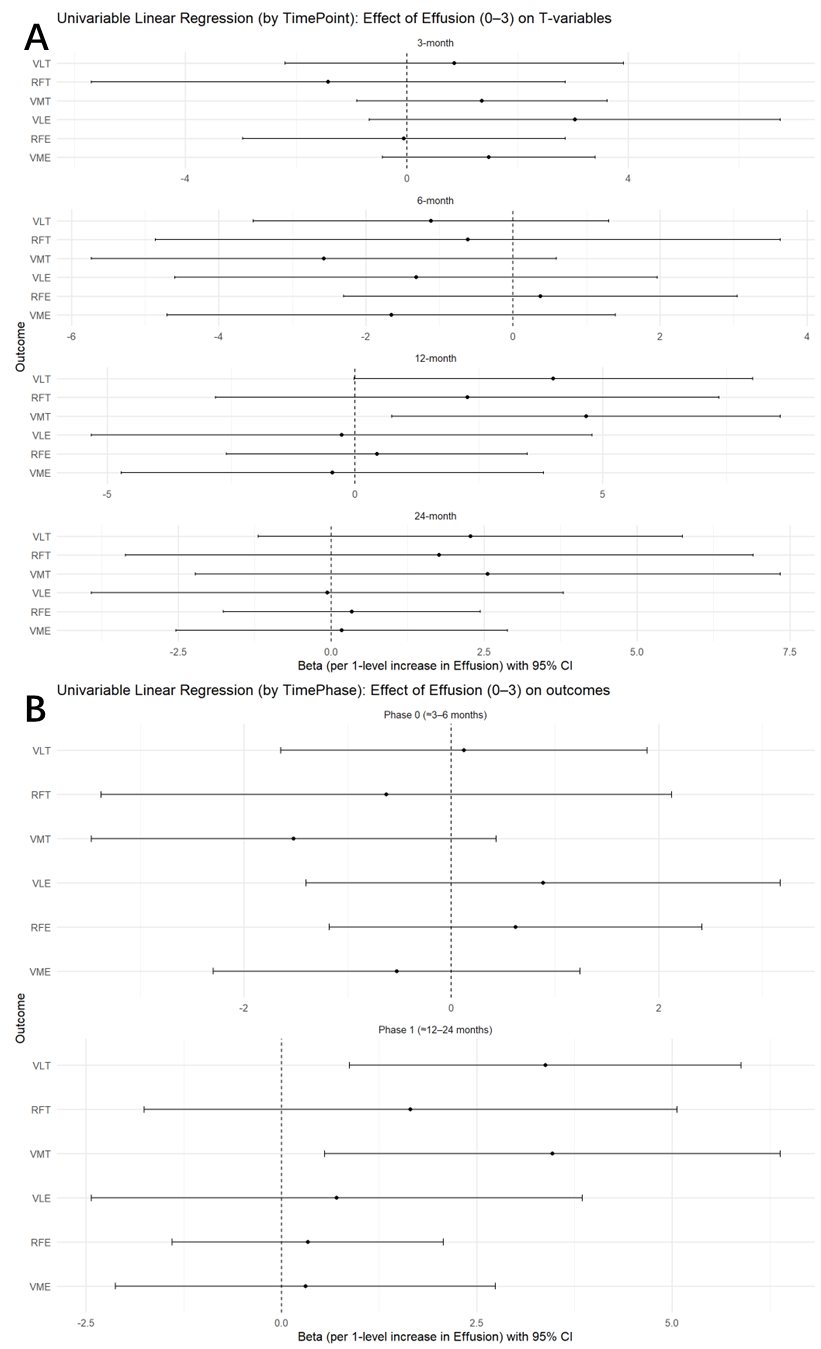


FIGURE S3. Univariate analysis between effusion and quadriceps strength, tension and activation. ISR, Insall-Salvati ratio*100%; BO, bisect offset*100%; PTA, patellar tilt angle; MVC, maximal voluntary contraction value; BW%, MVC normalized by body weight; VL, vastus lateralis; RF, rectus femoris, VM, vastus medialis; _T_, value of tension; _E_, value of activation.
